# Psychological therapies for the treatment of depression in chronic obstructive pulmonary disease

**DOI:** 10.1017/S1463423620000225

**Published:** 2020-10-06

**Authors:** Daksha Trivedi

**Affiliations:** Senior Research Fellow, Centre for Research in Public Health and Community Care, University of Hertfordshire, Hatfield, UK

**Keywords:** bronchitis, chronic obstructive pulmonary disease, depression, emphysema, intervention, psychological, therapy, treatment

**Review question:** To evaluate the effectiveness of psychological therapies for the treatment of depression in patients with chronic obstructive pulmonary disease.

## Relevance to primary care and nursing

Primary health care professionals including psychologists, psychiatrists and trained mental health nurses all have a pivotal role in the diagnosis and management of depression in adults including those with chronic physical health problems [National Institute for Health and Clinical Excellence (NICE), 2009, 2010].

## Characteristics of the evidence

This Cochrane review contains 13 randomised controlled trials involving 1500 participants (range 30-254) (Pollok et al., 2019). Included studies targeted adults (aged 40 years and over) diagnosed with chronic obstructive pulmonary disease (COPD) who presented with depressive symptoms at the time of recruitment into the trial. Although patients with other comorbidities (e.g., hypertension, cardiovascular disease, metabolic disease, asthma and mental disorders) were included, studies examining interventions specifically targeting anxiety were excluded. These included any form of psychological therapies compared with control groups having no intervention, education or a co-intervention [i.e., psychological therapy with pulmonary rehabilitation (PR) compared to PR alone] delivered in any format (e.g., group or one-on-one sessions, phone, email or internet-based) and in any setting. Interventions were delivered by trained health care professionals including nurses, physicians, psychologists and physiotherapists.

## Summary of key evidence

Follow-up ranged from 1 to 12 months. Pooled evidence was of very low quality overall judged using GRADE (The Grading of Recommendations Assessment, Development and Evaluation), mostly due to clinical heterogeneity and risk of bias.

Primary outcomes: depressive symptoms and adverse events.

Secondary outcomes: quality of life, dyspnoea, forced expiratory volume in one second (FEV_1_), exercise tolerance, hospital length of stay or readmissions and cost-effectiveness.

Continuous data were summarised as mean differences (MDs) or standardised MDs (SMDs), along with 95% CI from pooled trials. Significant effects are indicated below.

### Psychological therapy versus no intervention or education

Evidence from six trials (*n* = 764) showed that a cognitive behavioural therapy (CBT)-based intervention was effective in improving depressive systems compared to no intervention or to education in three trials (*n* = 507; SMD 0.23, 95% CI 0.06-0.41; *P* = 0.010). No adverse events were reported.

Evidence from three trials showed no significant effect of CBT-based programmes on the quality of life compared with no treatment. They did not measure other secondary outcomes.

Studies that compared psychological therapy with education reported inconclusive results for quality of life, dyspnoea and exercise tolerance. None of the studies measured FEV_1_, and one study (*n* = 222) measuring cost-effectiveness and hospital utilisation rates showed more significant reductions in hospital stay in the intervention group (42%) than in the education group (16%). The total number of bed days reduced by 61% in the psychological therapy group and increased by 18% in the education group. Intervention group showed a greater reduction in costs related to hospital admissions (GBP 23 263) compared with the education group (GBP 7439), with savings at 12-month follow-up of GBP 30 197.12 (269.62 per participant).

### Psychological therapy plus a co-intervention versus co-intervention alone

Evidence from two studies (*n* = 114) showed a greater reduction in depressive symptoms from psychological therapy combined with a PR programme compared with a PR programme alone (SMD 0.37, 95% CI −0.00 to 0.74; *P* = 0.05). Both studies had methodological limitations.

None of the studies measured adverse events. There was no significant effect on exercise tolerance and the studies did not report any other secondary outcomes.

## Implications for practice

The review did not find strong evidence that psychological therapies for patients with COPD and depression can reduce depressive symptoms in the long term. The quality of evidence was very low due to clinical heterogeneity and methodological limitations, which makes it difficult to interpret the findings.

## Implications for research

High-quality adequately powered trials are required to establish both effectiveness and cost-effectiveness of psychological therapies. The studies varied in delivery methods and format, and research needs to determine the mode of delivery that is effective for COPD-related depression. In addition, further studies are required to compare minimal interventions reported in this review with conventional psychological therapy to obtain adequate data on both primary and secondary outcomes.
